# A novel long-acting C5a-blocking cyclic peptide prevents sepsis-induced organ dysfunction via effective blockade of the inflammatory cascade

**DOI:** 10.1038/s41392-025-02457-8

**Published:** 2025-11-05

**Authors:** Zimiao Luo, Pengfei Luo, Haoyu Gu, Xiaoyan Hu, Shichu Xiao, Weiyue Lu, Zhaofan Xia

**Affiliations:** 1 Department of Burn Surgery, Burn Institute of PLA, The First Affiliated Hospital of Naval Medical University, Shanghai, 200433 China; 2 Department of Pharmaceutics, School of Pharmacy, Fudan University & Key Laboratory of Smart Drug Delivery (Fudan University), Ministry of Education and PLA, Shanghai, 201203, China; National Key Laboratory of Advanced Drug Formulations for Overcoming Delivery Barriers, Shanghai, 201203 China

**Keywords:** Drug delivery, Target validation, Drug development

## Abstract

Sepsis is a life-threatening syndrome characterized by dysregulated host responses to infection, leading to severe organ dysfunction and a high mortality rate. Reducing the incidence of sepsis is of paramount importance. Given that sepsis-associated drugs largely fail in clinical trials, in this project, we devised and validated a novel long-acting C5a-blocking cyclic peptide drug (Cp1) via phage screening technology to block the upstream “bottleneck molecule” C5a-mediated amplification cascade of the inflammatory response. In the early stage of infection, we utilized the efficient neutralization of Cp1 against C5a to effectively curb the “waterfall effect” of inflammatory factors and mitigate the progression to dysregulated systemic inflammation, thereby providing effective prevention and therapeutic intervention for sepsis. First, in vitro and in vivo studies collectively demonstrated the optimal binding affinity and blocking selectivity of Cp1. The excellent plasma stability of Cp1 further endows it with antibody-like systemic circulation. In the CLP-induced sepsis model, Cp1 significantly suppressed the expression of inflammatory factors and chemokines in both plasma and peritoneal lavage fluid (PLF). Additionally, Cp1 potently inhibited innate immune injury. Ultimately, after a single administration of Cp1, the CLP-induced septic mice presented a significant reduction in bacterial burden, evident amelioration of organ dysfunction, and notable prolongation of survival time. Overall, the novel cyclic peptide drug Cp1 developed in this study is a highly promising and cost-competitive therapeutic option for sepsis prophylaxis and therapy.

## Introduction

Sepsis is a life-threatening syndrome characterized by dysregulated host responses to infection, leading to severe organ dysfunction and high mortality rates.^1^ Sepsis and sepsis-associated multiple organ dysfunction syndrome (MODS) represent the leading cause of death in critically ill patients, with reported mortality rates ranging from 28 to 56%.^2^ In sepsis, uncontrolled hyperinflammation (“cytokine storm”) is one of the important initiating factors of MODS.^3^ Consequently, timely containment of excessive inflammatory responses is critical for determining the pathophysiological trajectory of sepsis.

At present, the clinical treatments for cytokine storm (CS) include cytokine and receptor antagonist therapy, hormone therapy, protease inhibitor therapy, blood purification therapy, etc.^4^ Monoclonal antibody-based “one-by-one” neutralization strategies exhibit limited therapeutic coverage due to redundant proinflammatory mediators while concurrently increasing susceptibility to secondary infections.^5^ The extensive side effects and risk of resistance limit the clinical use of glucocorticoids.^6^ The suboptimal therapeutic efficacy of CS in the clinic underscores an urgent unmet need for novel and effective pharmacologic interventions.

As a key upstream sensor and effector arm of innate immunity, the complement system amplifies the inflammatory cascade during sepsis via synergistic cross-talk with TLR.^7^ Excessive activation of the complement system is intricately related to the clinical outcomes of sepsis.^8,9^ Uncontrolled complement amplification exacerbates systemic hyperinflammation, promotes vascular hyperpermeability with consequent tissue edema and causes dysregulation of the clotting and fibrinolytic systems, collectively leading to multiorgan dysfunction.^10–12^ Concurrently, excessive activation of the complement system leads to a state of secondary hypocomplementaemia and increased risk of secondary infections.^13^ Therefore, inhibiting excessive complement activation early in infection can effectively regulate the inflammatory response, significantly improving sepsis outcomes.^14^

The effective modulation of complement activation in sepsis therapy may be achieved by mitigating its deleterious effects (cascade amplification) while preserving its protective function (bactericidal function). Upstream complement inhibitors (e.g., C3 inhibitors) broadly suppress complement activation but may increase the infection risk due to their comprehensive blockade.^15^ Pathway-specific complement inhibitors, while theoretically associated with a lower risk of infection, are only viable in diseases with defined complement pathway overactivation.^16^ As the most potent anaphylatoxin in the complement system (with 20-fold greater chemotaxis than C3a), C5a plays a central role in inflammatory responses and immune regulation.^8^ C5a functions through two identified receptors, C5aR1 (also termed CD88) and C5aR2 (also known as C5aL2). Current research indicates that C5aR1 is the primary proinflammatory receptor, whereas C5aR2 appears to be an anti-inflammatory receptor.^17^ C5a inhibitors demonstrate unique therapeutic advantages in the prevention and treatment of sepsis through their precise immunomodulatory effects. C5a inhibitors mitigate excessive inflammatory responses by selectively blocking the C5a‒C5aR axis while preserving other critical functions of the complement system, such as C3‒C3b‒dependent opsonization and MAC-mediated bacterial lysis.^18^

In the context of COVID-19, Vlaar et al. reported the first RCT showing increased survival of COVID-19 patients, leading to “urgent” FDA approval for vilobelimab (C5a-targeted monoclonal antibody) treatment in critically ill COVID-19 patients.^19^ Nevertheless, there remains an urgent need for novel C5a-targeted therapeutics to alleviate the economic burden of the extremely costly drugs on the market today and increase patient accessibility.

Plasma stability is an important prerequisite for effective targeted drug delivery and optimal therapeutic outcomes. The upregulated protease expression profile in septic plasma underscores the need for increased drug stability to maintain optimal therapeutic efficacy.^20,21^

**Fig. 1:**
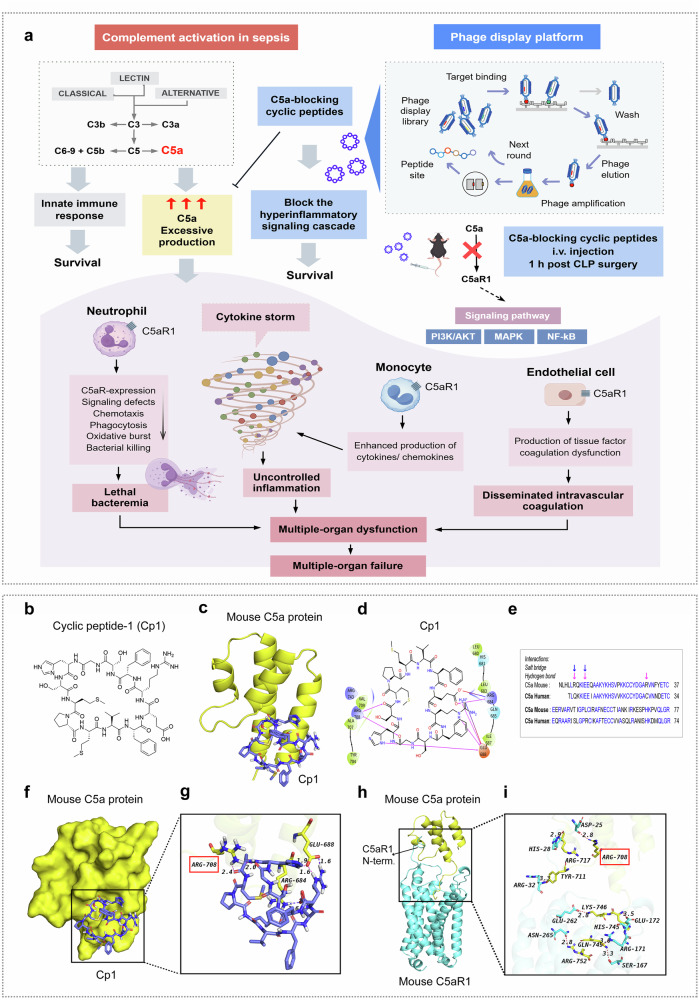
A novel C5a-blocking cyclic peptide drug, Cp1, for the preventive treatment of sepsis. **a** Schematic illustration of the discovery and mechanism of Cp1 for preventive treatment of sepsis. This figure illustrates the outcomes of excessive C5a production following complement activation in sepsis, such as the formation of a cytokine storm, functional paralysis of neutrophils, and coagulation dysfunction, ultimately leading to multiple organ dysfunction or even failure. In this project, we devised and validated a novel long-acting C5a-blocking cyclic peptide drug (Cp1) via phage screening technology to block the upstream C5a-mediated amplification cascade of the inflammatory response. In early infection, we utilized the efficient neutralization of Cp1 against C5a to effectively curb the “waterfall effect” of inflammatory factors and mitigate the progression to dysregulated systemic inflammation, thereby providing effective prevention and therapeutic intervention for sepsis. A schematic diagram is shown in the figure. **b** Molecular structure of Cp1 obtained via phage screening technology. **c** Cartoon diagram of the binding of Cp1 to the mouse C5a protein. **d** 2D diagram of Cp1 binding to the mouse C5a protein. **e** Sequence alignment between mouse C5a and human C5a. The interactions involved are summarized with magenta (hydrogen bond) or blue (salt bridge) arrows. **f** Surface diagram of the binding of Cp1 to the mouse C5a protein. **g** 3D view of the binding of Cp1 to the mouse C5a protein. Cp1 is shown in purple. The mouse C5a protein is shown in yellow. Hydrogen bond interactions are shown as magenta dashed lines. The numbers indicate the hydrogen bond length. Salt–bridge interactions are shown as blue dashed lines. **h** Cartoon diagram of the mouse C5a/C5aR1 binding mode. **i** 3D view of the mouse C5a/C5aR1 binding mode: the mouse C5aR1 protein is shown in blue. The mouse C5a protein is shown in yellow. Hydrogen bond interactions are shown as red dashed lines

In this study, we devised and validated a novel and cost-competitive long-acting C5a-blocking cyclic peptide drug (Cp1) via phage screening technology to block the upstream “bottleneck molecule” C5a-mediated amplification cascade of the inflammatory response (Fig. 1a, b). In early infection, we aimed to harness the neutralizing effects of Cp1 against C5a to effectively curb the “waterfall effect” of inflammatory factors and mitigate the progression to dysregulated systemic inflammation, thereby providing effective prevention and therapeutic intervention for sepsis. We revealed the structural basis of the Cp1‒C5a interactions via molecular docking analysis. We systematically evaluated the binding affinity and blocking specificity of Cp1 to C5a in vitro and in vivo and assessed its suppressive effects on inflammatory factors and chemokines in the plasma and PLF. Furthermore, we investigated the efficacy of Cp1 in mitigating innate immune dysfunction, protecting against organ injury, and prolonging survival time in septic mice. Additionally, RNA-seq analysis was employed to confirm the specific inhibition of C5a signaling by Cp1 at the transcriptional level.

## Results

### Molecular docking and binding mode analysis reveals the mouse C5a protein (mC5a)-Cp1 interaction patterns

To elucidate the structural basis of the enhanced binding affinity and specificity of Cp1 compared with those of K1 (the linear form of Cp1), we performed molecular docking and binding mode analyses to reveal the protein‒cyclic peptide interaction patterns. The results of molecular docking revealed a cartoon diagram of mC5a‒Cp1 binding (Fig. 1c), 2D diagram (Fig. 1d), surface diagram (Fig. 1f) and 3D diagrams (Fig. 1g), respectively. The C skeleton of the mC5a protein is shown in yellow, with N atoms colored blue, O atoms in bright red, H atoms in white, and the Cp1 moiety represented as a purple stick model (Fig. 1g). The hydrogen bonding interactions are shown as magenta dashed lines. The salt-bridge interactions are shown as blue dashed lines. (Fig. 1g). Sequence alignment between mouse C5a and human C5a highlighted residues potentially involved in Cp1 selectivity (Fig. 1e). As shown in Fig. 1d, g, Cp1 formed six hydrogen bonds and two salt bridges with the mC5a protein. Specifically, the multiple ketone groups in Cp1 as hydrogen bond receptors formed two hydrogen bonds with ARG708 (mC5a), with distances of 2.0 Å and 2.4 Å, respectively. The nitrogen on imidazole (Cp1) as the hydrogen bond donor formed a hydrogen bond with GLU688 (mC5a) at a distance of 1.9 Å. As a hydrogen bond acceptor, the carboxyl oxygen in Cp1 formed a hydrogen bond with ARG684 (mC5a) at a distance of 1.6 Å. The multiple amino groups (Cp1) acted as hydrogen bond donors and formed two hydrogen bonds with GLU688 (mC5a), with a distance of 1.6 Å. In addition, Cp1 also formed two salt bridges with ARG684 (mC5a) and GLU688 (mC5a) (Fig. 1d, g). The molecular docking results of mC5a protein-K1 binding cartoon diagram are shown in Supplementary Figure 1. The binding sites of K1 and Cp1 to mC5a clearly differed substantially. The hydrogen bond interactions formed between K1 and mC5a were relatively weak, with hydrogen bond lengths of 2.6 Å, 3.1 Å, 2.9 Å, 3.0 Å, 2.9 Å and 2.9 Å, respectively (Supplementary Fig. 1). We further analyzed the binding mode of mC5a/C5aR1 (Fig. 1h, i). The 3D diagram indicated that seven hydrogen bonds formed between mC5a and C5aR1. Among them, two stronger hydrogen bonds (2.0 Å and 2.4 Å) (Fig. 1g) formed by Cp1 and ARG708 (mC5a) may disturb the interaction (2.8 Å) (Fig. 1h) between ARG708 (mC5a) and ASP25 (mC5aR1). In summary, Cp1 exhibited stronger interactions with mC5a than did K1. The distinct binding site of Cp1 more effectively blocked mC5a-C5aR1 interactions, which provides a structural rationale for the significantly increased binding affinity of Cp1 compared with that of K1. In the cyclization strategy, the enhanced electrostatic shielding effects are mediated by cyclic peptide‒protein interactions, and the hydrophobic regions in C5a synergistically minimize the solvent accessibility of Cp1, thereby increasing its plasma stability.

**Fig. 2:**
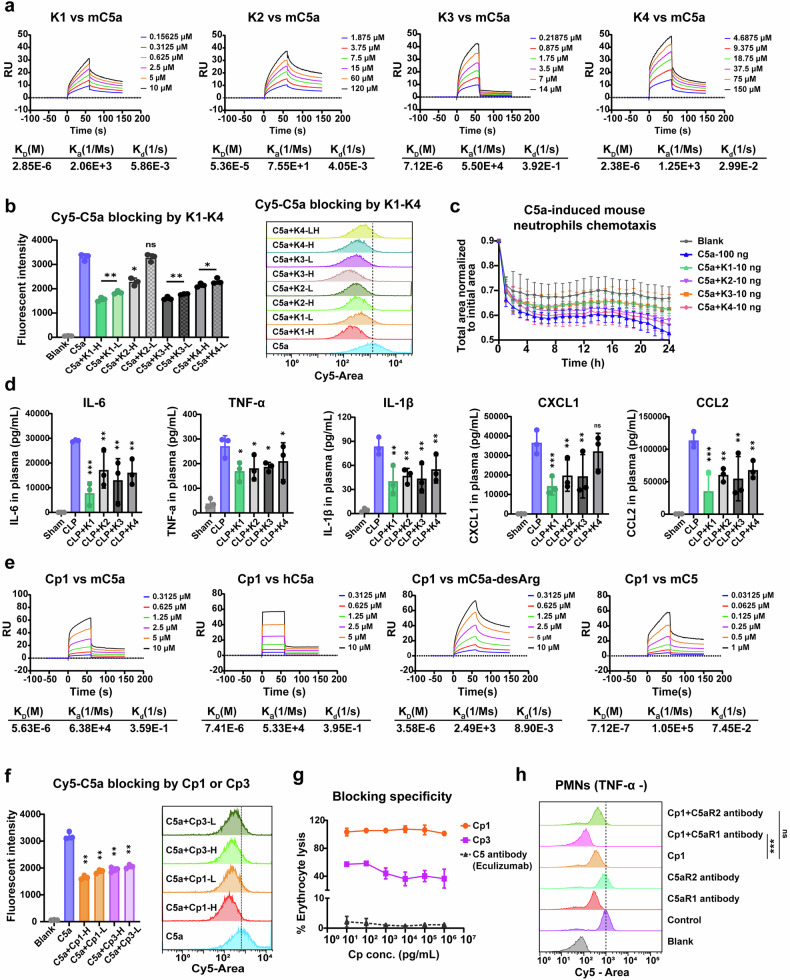
Validation of the binding affinity and blocking specificity of C5a-blocking Cp1. **a** SPR analysis of the mouse C5a protein with linear peptides K1, K2, K3 or K4. **b** Flow cytometry analysis of competitive inhibition assays for C5a-blocking linear peptides K1, K2, K3, and K4 at high (1 μg/mL) or low (10 ng/mL) concentrations, *n* = 3. **c** The inhibitory efficiency of C5a-blocking linear peptides K1, K2, K3 and K4 on mouse neutrophil migration determined by chemotaxis assay at a dose of 10 ng per well, *n* = 5. **d** The in vivo inhibitory efficiency of C5a-blocking linear peptides K1, K2, K3 and K4 on plasma proinflammatory cytokines and chemokines determined by ELISA at a dose of 100 μg/20 g via i.v. injection, *n* = 3. **e** SPR analysis of cyclic-peptide Cp1 with mouse C5a, human C5a, mouse C5a-desArg or mouse C5. **f** Flow cytometry analysis of competitive inhibition assays for Cp1 and Cp3 at high (1 μg/mL) or low (10 ng/mL) concentrations, *n* = 3. **g** Cyclic peptide Cp1, Cp3 or anti-C5 antibody (eculizumab)-induced erythrocyte hemolysis rate quantified by hemoglobin absorbance at 540 nm, *n* = 3. **h** Validation of Cp1-specific blockade of C5a-C5aR1 detected by flow cytometry. PMNs (without TNF-α stimulation) were preincubated with C5aR1-antibody, C5aR2-antibody, Cp1, Cp1 + C5aR1-antibody or Cp1 + C5aR2-antibody at room temperature for 0.5 h, followed by incubation with Cy5-C5a at 4 °C for 0.5 h in the dark, *n* = 3. The data are presented as the means ± SDs. *P < 0.05, **P < 0.01, ***P < 0.001

**Fig. 3:**
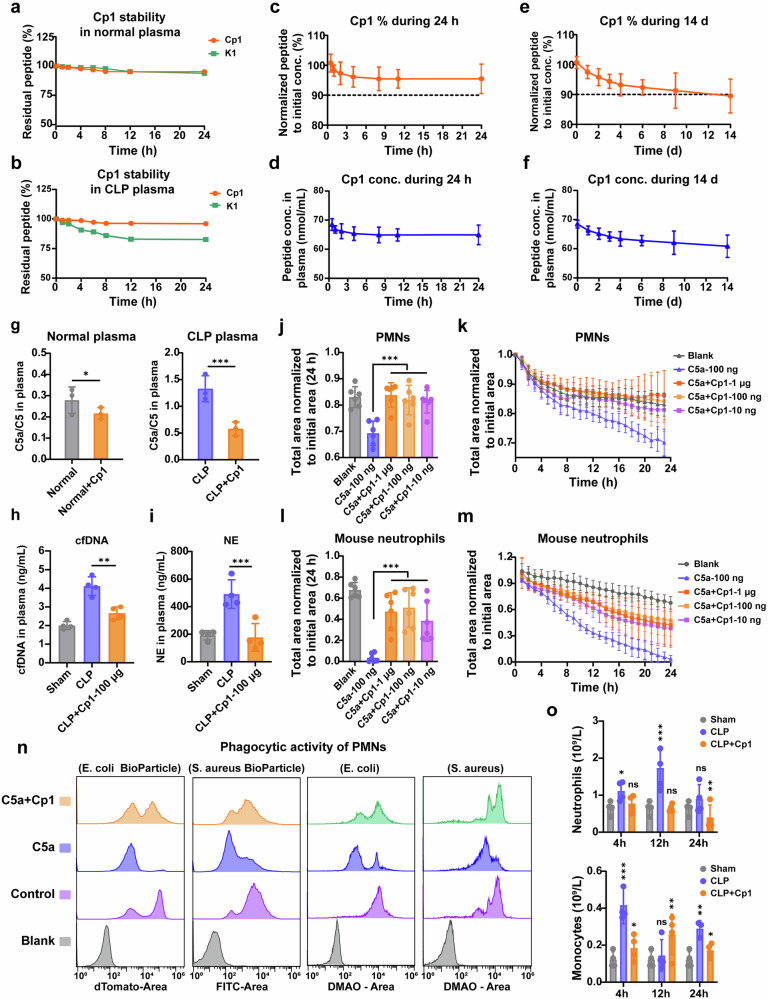
Plasma stability and pharmacokinetics study of C5a-blocking Cp1 and the functional modulation of PMNs by Cp1-mediated C5a blockade. **a**, **b** Stability of C5a-blocking linear peptide K1 and cyclic peptide Cp1 in 50% (v/v) normal plasma or CLP plasma for 24 h, *n* = 3. **c**, **d** Plasma residual Cp1 percentage‒time curves or plasma Cp1 concentration‒time curves during the first 24 h after intravenous administration (100 μg of Cp1), *n* = 5. **e**, **f** The plasma residual Cp1 percentage‒time curves or the plasma Cp1 concentration‒time curves at 14 days after intravenous administration (100 μg of Cp1), *n* = 5. **g** The ratio of plasma C5a to C5 in normal mice or CLP model mice was determined via ELISA after Cp1 (100 μg/20 g) administration, *n* = 3. **h** The quantitative results of cfDNA in plasma 24 h after CLP surgery after Cp1 treatment (100 μg/20 g), *n* = 4. **i** The quantitative results of neutrophil elastase (NE) in plasma 24 h after CLP surgery after Cp1 treatment (100 μg/20 g), *n* = 4. The quantitative results of the chemotaxis assay for PMNs (**j**) and mouse neutrophils (**l**) at 24 h, *n* = 6. The inhibitory effects of Cp1 on PMN (**k**) or mouse neutrophil (**m**) migration were determined via a chemotaxis assay with a Cp1 concentration gradient from 10 ng to 1 μg per well, *n* = 6. **n** The protective effects of Cp1 on C5a-induced phagocytic impairment in PMNs were quantified via flow cytometry, *n* = 3. PMNs (2 × 10^5^) were prestimulated with 100 ng of hC5a or hC5a/Cp1 complexes for 6 h. PMNs were further incubated with 20 μL of Red *E. coli* BioParticles™ or Green *S. aureus* BioParticles™ (Invitrogen, USA) at 37 °C for 0.5 h in the dark. PMNs were incubated with DMAO-labeled *E. coli* or *S. aureus* at an MOI of 10 at 37 °C for 0.5 h in the dark, followed by gentamicin treatment (*n* = 3). **o** Absolute numbers of neutrophils or peripheral monocytes in CLP model mice were detected via routine blood tests at predetermined time points (4 h, 12 h and 24 h) after intravenous administration of Cp1 (100 μg/20 g), *n* = 4. The data are presented as the means ± SDs. *P < 0.05, **P < 0.01, ***P < 0.001

### Discovery of novel C5a-blocking peptides

The mouse C5a protein was used as the antigen to screen specific binding sequences from a 12-peptide library via a phage display system. After three rounds of screening, eighty monoclonal clones were selected for sequencing, and seven specific sequences were obtained. The K_D_ values of K1-K5 (5.94E-6, 1.73E-5, 1.10E-4, 8.50E-4 and 1.09E-2) were preliminarily determined via a biolayer interferometry (BLI) rapid affinity assay. We further validated the binding affinity of the linear peptides K1-K4 for mouse C5a via an SPR assay. The K_D_ values of the linear peptides K1, K2, K3 and K4 were 2.85E-6, 5.36E-5, 7.12E-6 and 2.38E-6, respectively (Fig. 2a). K1 and K3 exhibited superior water solubilities to those of K2 and K4, enhancing their suitability for systemic administration in septic mice.

### Validation of the blocking effects of linear peptides

Mouse neutrophils (highly expressing C5aR1 + ) were selected for a competitive binding assay to verify the ability of the peptides to block the Cy5-C5a protein. K1 and K3 demonstrated superior C5a blockade to K2 and K4 in the flow cytometric assay (Fig. 2b). In the C5a-triggered neutrophil chemotaxis assay, the real-time total residual cell areas were monitored with an IncuCyte ZOOM^®^ Live-Cell Imaging System (Fig. 2c) further confirmed the stronger blocking capacity of K1 and K3 than that of K2 and K4. The plasma inflammatory cytokine and chemokine levels in septic mice were quantified via an ELISA 24 h after administration, which revealed that K1 and K3 exhibited better in vivo anti-inflammatory activity than did K2 and K4 (Fig. 2d). Given these findings, we selected K1 and K3 for further validation.

### The cyclization strategy improves the binding affinity and blocking specificity as well as the plasma stability of linear peptides

To improve the binding affinity, structural stability and in vivo blocking efficacy of the linear peptides K1 and K3, we designed cyclic variants through head‒tail cyclization, named Cp1 and Cp3, respectively. The binding affinities of Cp1 and Cp3 for the mC5a protein were measured via SPR, and the K_D_ value was 5.63E-6 (Fig. 2e) and 3.23E-6 (Supplementary Fig. 2), respectively. The binding kinetics of Cp1 to human C5a (7.41E-6) were similar to those of mC5a (Fig. 2e). Moreover, Cp1 exhibited binding affinity for mouse C5a-desArg (3.58E-6) and mouse C5 (7.12E-7) (Fig. 2e) as well. The in vitro competitive binding assay further validated the blocking efficacy of Cp1 and Cp3 on the mC5a protein (Fig. 2f).

A hemolysis assay was employed to investigate the binding specificity of Cp1. As a positive control, the C5 antibody eculizumab almost completely inhibited hemolysis. The nearly 100% hemolysis of sheep erythrocytes in the Cp1 group demonstrated that the binding of Cp1 to C5a does not interfere with C5 cleavage or C5b-dependent terminal MAC formation (Fig. 2g). In contrast, the partial erythrocyte lysis observed in the Cp3 group suggested a relatively weaker binding specificity of Cp3 to C5a than to Cp1 (Fig. 2g). We also investigated the specificity of Cp1 in blocking the C5a‒C5aR1 axis. The flow cytometry results in Fig. 2h and Supplementary Fig. 3 revealed that the anti-C5aR1 antibody significantly reduced Cy5-C5a binding to both resting and activated PMNs in the presence of Cp1, confirming the Cp1-selective blockade of the C5a‒C5aR1 interaction.

The plasma stability of Cp1 and K1 was assessed in 50% (v/v) plasma from normal mice or CLP model mice at 37 °C. The HPLC results revealed that Cp1 and K1 exhibited comparable stabilities in normal plasma, while the cyclization strategy obviously improved the K1’ plasma stability in CLP plasma, with over 95% of the Cp1 content remaining after 24 h of incubation (Fig. 3a, b). The pharmacokinetic study of Cp1 (100 μg/20 g) was subsequently carried out in normal mice, with plasma concentrations of Cp1 quantified by HPLC for 14 days. Cp1 exhibited slow elimination within 24 hours (Fig. 3c, d). Surprisingly, 90% of the initial concentration of Cp1 in the plasma (68 nmol/mL) was retained 14 days after administration (Fig. 3e, f).

### Cp1 treatment effectively inhibits the C5a-driven recruitment and activation of neutrophils

We evaluated the in vivo neutralization of Cp1 against C5a by measuring the plasma C5a/C5 ratio in normal mice or CLP model mice. The decreased ratio of C5a/C5 after Cp1 treatment, especially in the CLP model mice, demonstrated the effective neutralizing effects of Cp1 on mouse C5a (Fig. 3g).

Neutrophils serve as pivotal effector cells in the pathophysiological cascade of sepsis, and their functional involvement spans the entire disease course. The complement cleavage product C5a profoundly modulates neutrophil activation, chemotaxis and phagocytosis via the C5a‒C5aR axis. We quantified the plasma cell-free DNA (cfDNA) and neutrophil elastase (NE) levels after Cp1 administration (100 μg/20 g) via qPCR and ELISA methods, respectively. The quantitative results in Fig. 3h, i demonstrated that Cp1 administration significantly decreased the plasma cfDNA ( ↓ 68%) and NE ( ↓ 100%) levels, providing direct evidence that Cp1 suppresses neutrophil activation and respiratory bursts. The percent inhibition was calculated relative to the Sham group (set as 0%).

The chemotaxis assay in this study was monitored and quantified by the IncuCyte ZOOM® Live-Cell Imaging System. The y-axis label “total area normalized to initial area in each group” in Fig. 3k, m represent the ratio of the total cell area at each time point to the initial area (0 h) within the same field of view. A higher ratio indicates lower cell migration activity. As shown in Fig. 3k, m, Cp1 dose-dependently suppressed C5a-driven recruitment in both PMNs and mouse neutrophils, indicating the effective blockade of Cp1 against C5a. The Fig. 3j and l displayed the 24 h quantification results of Fig. 3k and m, respectively.

We initially quantified the phagocytic function of PMNs via flow cytometry utilizing Red *E. coli* BioParticles™ and Green *S. aureus* BioParticles™. We subsequently assessed PMN phagocytosis via live *E. coli* (a gram-negative organism) and *S. aureus* (a gram-positive organism). Following 6 h of C5a stimulation, we observed an obvious reduction in the fluorescence signal triggered by C5a in both the bioparticle and live bacterial assays, which could be attenuated by preincubation with Cp1 (Fig. 3n). Under an equivalent dose of C5a, PMNs exhibited a smaller reduction in phagocytic efficiency against *S. aureus* than against *E. coli*. This phenomenon may be attributed to the fact that the clearance of *S. aureus* relies on C5a-insensitive pathways, whereas the elimination of *E. coli* is highly dependent on the complement-ROS axis, which is disrupted by C5a. These results collectively demonstrated that Cp1 effectively mitigated C5a-induced phagocytic impairment in PMNs.

### Cp1 treatment significantly suppresses the expression of inflammatory factors and chemokines in both the plasma and peritoneal lavage fluid (PLF) of CLP-induced septic mice

We conducted a hematologic analysis to illustrate the dynamic changes in neutrophils and monocytes across different experimental groups after CLP surgery. As shown in Fig. 3o, The number of circulating neutrophils in the CLP group increased by 70% (compared with that in the sham group) at 4 h and peaked at 12 h (increased by 265% compared with that in the sham group). In contrast, compared with the CLP group, the Cp1-treated group showed no significant increase in the number of circulating neutrophil bursts, with a 63% reduction in the absolute neutrophil count at 12 h compared with that in the CLP group. The absolute monocyte count in the CLP group reached its first peak at 4 h (increased by 148% compared with that in the sham group), whereas the Cp1-treated group displayed a delayed peak (increased by 69% compared with that in the sham group) at 12 h, with a 38% reduction in the absolute monocyte count compared with the 4-h peak value in the CLP group.

**Fig. 4:**
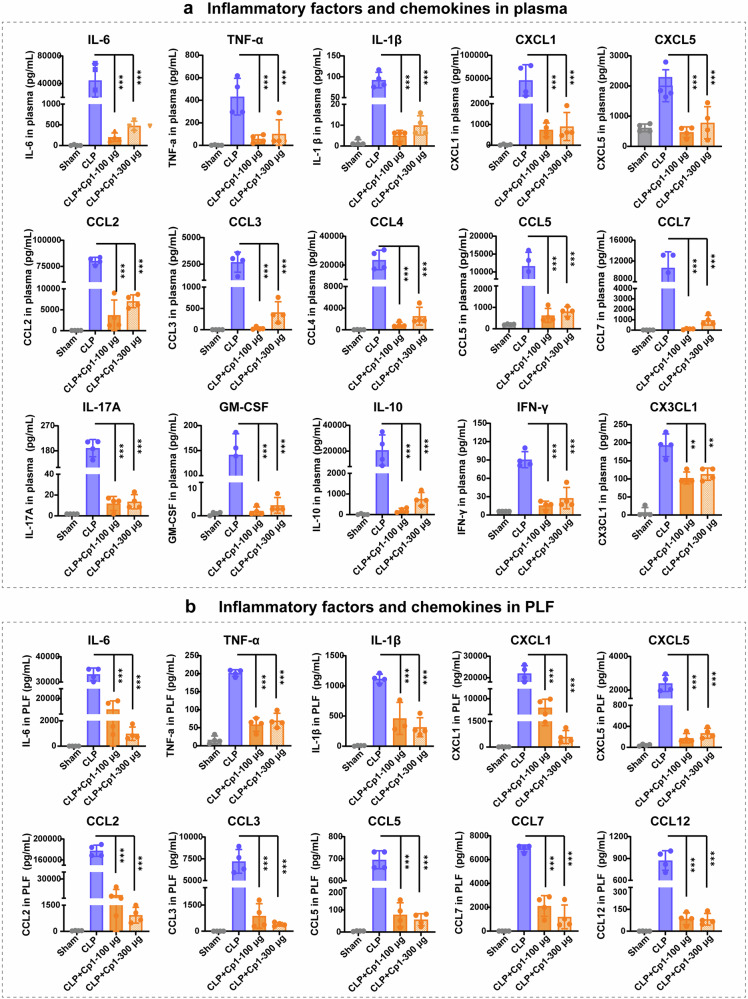
Inhibitory effects of C5a-blocking Cp1 on inflammatory factors and chemokines in both plasma and peritoneal lavage fluid (PLF) from CLP-induced septic mice. **a** Inflammatory factors and chemokines in plasma determined by the Luminex assay 24 h after CLP surgery with different doses of Cp1 administration (100 or 300 μg/20 g) via i.v. injection, *n* = 4. **b** Inflammatory factors and chemokines in the PLF determined by the Luminex assay 24 h after CLP surgery with different doses of Cp1 administration (100 or 300 μg/20 g) via i.v. injection, *n* = 4. The data are presented as the means ± SDs. *P < 0.05, **P < 0.01, ***P < 0.001

The potential inhibitory effects of Cp1 on the C5a-mediated amplification cascade in the inflammatory response were systematically evaluated through quantitative analysis of proinflammatory cytokines and chemokines in both systemic circulation (plasma) and local inflammatory sites (peritoneal lavage fluid). As shown in Fig. 4a, 24 h after CLP surgery, 100 μg/20 g Cp1 treatment resulted in a substantial reduction in plasma proinflammatory cytokine levels compared with those in the CLP group, with 99%, 86% and 94% decreases in the IL-6, TNF-α and IL-1β levels, respectively. Additionally, after Cp1 administration, the levels of the monocyte chemokines CCL2, CCL3, CCL4, CCL5 and CCL7 decreased 21.7-, 81.9-, 24.8-, 18.6- and 102.3-fold, respectively. Similarly, the levels of the neutrophil chemokines CXCL1 and CXCL5 were also decreased by 61.7-fold and 4.3-fold, respectively. Moreover, significantly decreased plasma IL-17A, IFN-γ, GM-CSF, and CX3CL1 levels were observed in the Cp1-treated groups. Similarly, 100 μg/20 g Cp1 treatment also significantly attenuated the levels of the proinflammatory cytokines IL-6 ( ↓ 73%), TNF-α (↓77%) and IL-1β ( ↓ 62%) as well as the chemokines CXCL1 ( ↓ 75%), CXCL5 ( ↓ 94%), CCL2 ( ↓ 96%), CCL3 ( ↓ 87%), CCL5 ( ↓ 89%), CCL7 ( ↓ 69%) and CCL12 ( ↓ 90%) in PLF (Fig. 4b). The percent inhibition was calculated relative to the Sham group (set as 0%). Moreover, the significantly stronger suppression of plasma cytokines by Cp1 than K1 validated the in vivo benefits of the cyclization design. The administration of K1 (100 μg/20 g) resulted in 3.7-fold, 1.5-fold and 2.0-fold reductions in the plasma IL-6, TNF-α and IL-1β levels, respectively (Fig. 2d). In contrast, Cp1 treatment (100 μg/20 g) demonstrated markedly greater efficacy than did K1, resulting in 220.5-fold, 7.5-fold and 18.6-fold reductions in the same cytokines, respectively (Fig. 4a). High-dose 300 μg of Cp1 also effectively suppressed the expression of inflammatory factors and chemokines in both the plasma and PLF (Fig. 4). Collectively, these results confirmed the effective suppression of inflammatory factors and chemokines by Cp1 in septic mice. Given that the preliminary titrations indicated that 100 μg was the lowest effective dose with significant pharmacological activity, we selected 100 μg for subsequent animal experiments. These results from Figs. 3o and 4 collectively demonstrated that Cp1 administration effectively mitigated early inflammatory responses by blocking C5a-induced recruitment and hyperactivation of innate immune cells.

### Cp1 treatment effectively prevents organ dysfunction in CLP-induced septic mice

**Fig. 5:**
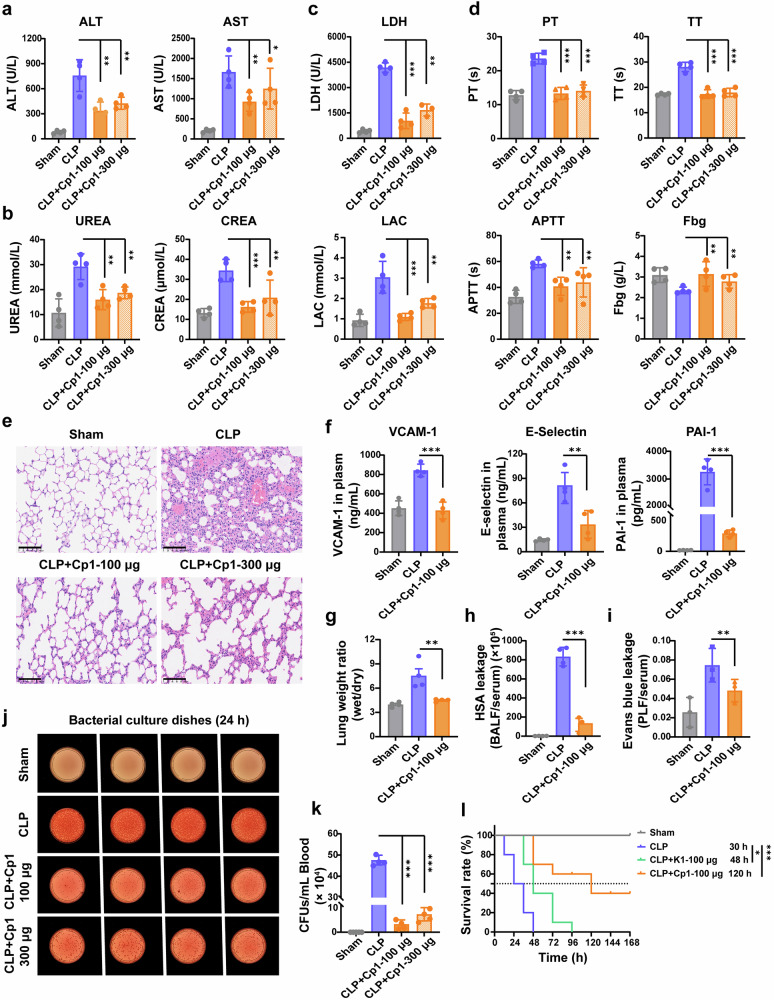
Treatment with C5a-blocking Cp1 effectively prevented organ dysfunction and promoted bacterial clearance in CLP-induced septic mice. Liver (**a**) and kidney (**b**) functions were assessed in CLP model mice 24 h post-CLP surgery after Cp1 (100 μg/20 g) administration, *n* = 4. **c** Plasma LDH or LAC levels in CLP model mice 24 h post-CLP surgery after Cp1 (100 μg/20 g) administration, *n* = 4. **d** Coagulation function parameters in CLP model mice 24 h post-CLP surgery after Cp1 (100 μg/20 g) administration, *n* = 4. **e** H&E staining results of lung tissue pathological sections collected 24 h post-CLP surgery, *n* = 3. The scale bar represents 100 μm. **f** The plasma VCAM-1, E-selectin or PAI-1 levels 24 h post-CLP surgery after Cp1 (100 μg/20 g) administration, *n* = 4. **g** The wet/dry weight ratio of lung tissue in each group 24 h post-CLP surgery, *n* = 4. **h** The capillary leakage in each group was quantified by the ratio of HSA level in BALF to serum 24 h post-CLP surgery, *n* = 4. **i** The capillary leakage in each group was quantified by the ratio of Evans blue in PLF to serum 24 h post-CLP surgery, *n* = 3. **j** The figures of 24 h bacterial culture dishes with whole blood from each group collected 24 h post-CLP modeling, *n* = 4. **k** The quantitative results of CFUs/mL in blood in each group 24 h post-CLP modeling, *n* = 4. **l** The Kaplan–Meier survival curves of CLP model mice during the first 7 days after cyclic peptide (Cp1) or linear peptide (K1) administration (100 μg/20 g), *n* = 10. The data are presented as the means ± SDs. *P < 0.05, **P < 0.01, ***P < 0.001

In sepsis, high mortality is attributed primarily to damage to and acute failure of vital organs. As shown in Fig. 5a, treatment with Cp1 effectively mitigated the increase in the serum alanine aminotransferase (ALT) and aspartate aminotransferase (AST) levels, suggesting that Cp1 has protective effects on liver function. Compared with untreated CLP mice, Cp1-treated mice also presented significantly lower serum levels of UREA (urea) and CREA (creatinine), biomarkers of kidney injury (Fig. 5b). Elevated serum LDH (lactate dehydrogenase) and LAC (lactic acid) levels usually indicate tissue injury in the clinic. The marked reduction in LDH and LAC levels observed in the Cp1-treated group underscored the potent organ-protective efficacy of Cp1 (Fig. 5c).

In sepsis, complement–coagulation crosstalk drives disease progression. Sepsis is often associated with consumptive coagulopathy, indicating a loss of control of the clotting and fibrinolytic systems, such as elevated PT (prothrombin time) or APTT (activated partial thromboplastin time), prolonged TT (thrombin time) and decreased Fbg (fibrinogen).^22^ Targeted blockade of C5a would provide dual therapeutic benefits—simultaneously suppressing excessive inflammation and mitigating coagulation dysfunction in sepsis. As shown in Fig. 5d, targeted blockade of C5a by Cp1 attenuated the sepsis-induced changes in the clotting and fibrinolytic systems.

Furthermore, we evaluated the protective effect of Cp1 against lung injury in septic mice. Cp1 treatment significantly ameliorated sepsis-induced pulmonary vascular leakage, reducing hallmark pathological features such as alveolar hemorrhage, septal thickening, and inflammatory infiltration (Fig. 5e). Compared with those in the CLP group, the levels of the circulating endothelial injury biomarkers VCAM-1, E-selectin and PAI-1 decreased by 100%, 71% and 93%, respectively, after Cp1 administration (Fig. 5f). Additionally, compared with the untreated CLP group, the Cp1-treated group exhibited notable improvements in lung endothelial integrity, as evidenced by a reduction in the wet-to-dry weight ratio (W/D ratio) ( ↓ 85%), decreased HAS leakage (BALF/Serum) ( ↓ 83%) and lower Evans blue leakage (PLF/Serum) ( ↓ 54%), all of which collectively demonstrated the protective effects of Cp1 on the endothelium (Fig. 5g–i).

### Cp1 treatment effectively controls the bacterial burden and significantly prolongs the survival of CLP-induced septic mice

To assess the therapeutic efficacy of Cp1 in modulating systemic bacterial dissemination during sepsis, we performed quantitative bacterial cultures of peripheral blood samples from CLP model mice. The 24 h bacterial culture dishes and CFU counting results are shown in Fig. 5j, k. Compared with the CLP treatment, the 100 μg and 300 μg Cp1 treatments effectively reduced the bacterial burden in septic mice by 93% and 86%, respectively, indicating that early Cp1-mediated immunomodulation, which is based on the effective blockade of C5a, restored immune equilibrium and visibly enhanced bacterial clearance in septic mice.

Finally, after a single administration of Cp1 or K1 (100 μg/20 g), we analyzed the survival of septic mice via the Kaplan–Meier method. Animal mortality was determined by the presence of confirmed death. As shown in Fig. 5l, the 7-day survival rates in the CLP, CLP + K1 and CLP+Cp1 groups were 0%, 0% and 60%, respectively. The median survival times of septic mice in the CLP, CLP + K1 and CLP+Cp1 groups were 30, 48, and 120 h, respectively. The significant increase in survival of septic mice in the CLP+Cp1 group demonstrated the effective intervention with Cp1, suggesting that C5a blockade is a viable strategy for sepsis prevention and therapy.

### Cp1 treatment achieves effective clearance of secondary infection

**Fig. 6:**
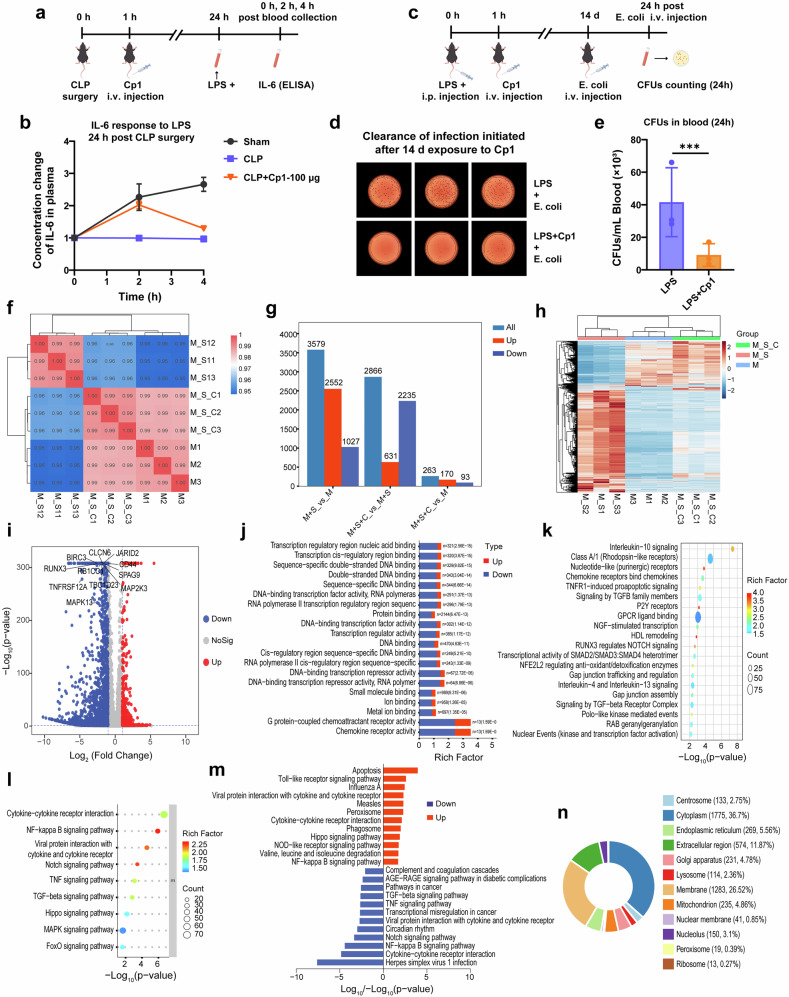
Response of Cp1-treated mice to secondary infection and Cp1 treatment-induced changes in the mRNA levels of peripheral mononuclear cells. **a** Experimental timeline of CLP-treated mice receiving LPS stimulation. **b** Plasma IL-6 response to LPS stimulation in each group 24 h post-CLP surgery, *n* = 4. **c** Experimental timeline of LPS-treated mice receiving live bacterial infection after 14 d of exposure to Cp1 (100 μg/20 g), *n* = 3. **d** Clearance results of infection initiated after 14 d of exposure to Cp1 are presented in 24 h blood bacteria culture dishes, *n* = 3. **e** CFU count results of Fig. 6d, *n* = 3. The data are presented as the means ± SDs. *P < 0.05, **P < 0.01, ***P < 0.001. **f** Sample correlations among the three groups. **g** Statistical bar graph of differentially expressed genes (DEGs) between the Cp1-treated and untreated groups. **h** Cluster analysis of mRNA samples among the three groups. **i** Volcano plot of DEGs between the Cp1-treated and untreated groups. **j** The top 20 molecular function (MF) terms significantly enriched in DEGs between the Cp1-treated and untreated groups, as determined via GO analysis. **k** Bubble plot of REACTOME pathway enrichment between the Cp1-treated and untreated groups. **l** Bubble plot of the top 30 enriched metabolism-associated genes in the KEGG pathway between the Cp1-treated and untreated groups. **m** The top 12 upregulated and downregulated genes identified via KEGG pathway enrichment analysis between the Cp1-treated and untreated groups. **n** The subcellular localization of DEGs based on GO enrichment between the Cp1-treated and untreated groups. The control group, C5a-stimulated group and Cp1-treated group are represented as the M, M + S and M + S + C groups, respectively, *n* = 3. The screening criteria for DEGs were a |log2FoldChange | ≥ 1 and a *P* < 0.05

To investigate the impact of short-term drug exposure on the secondary infection response. We compared the LPS responsiveness of the Cp1 group with that of the CLP control group 24 h post-CLP surgery (Fig. 6a). As shown in Fig. 6b, at 2 h post-LPS stimulation, the Cp1 group presented obviously increased IL-6 levels compared with those of the CLP control group, suggesting that Cp1 exposure beneficially modulated the LPS responsiveness of CLP-treated mice during the intermediate phase (24 h) of sepsis.

To evaluate the impact of long-term drug exposure on the secondary infection response, LPS model mice received intravenous live bacterial injection after 14 d of exposure to Cp1 (Fig. 6c). The encouraging CFU counting results are shown in Fig. 6d, e. Compared with the untreated control, the Cp1 treatment still effectively cleared new infections initiated after prolonged exposure (i.e., 14 days) to Cp1.

### Cp1 treatment blocks and reshapes the C5a-induced alterations in gene expression in peripheral monocytes

To investigate the regulatory effects of Cp1-targeted blockade of C5a-C5aR1 on downstream signaling pathways, RNA-seq analysis was performed in human peripheral blood mononuclear cells (PBMCs) after C5a stimulation. The screening criteria for differentially expressed genes were a |log2FoldChange | ≥ 1 and a *P* < 0.05. As shown in Fig. 6f, the samples in all the groups exhibited high intragroup consistency. C5a stimulation yielded 2552 upregulated differentially expressed genes (DEGs) in PBMCs (Fig. 6g). However, 2235 DEGs were notably downregulated after Cp1 preincubation (Fig. 6g), resulting in gene expression profiles of the Cp1-treated group that were comparable to those of the untreated controls (Fig. 6h). The volcano plot further annotated the key downregulated genes following Cp1 preincubation, which were functionally associated with inflammatory regulation, proinflammatory cytokine secretion, increased immune cell recruitment, and prolonged leukocyte survival (Fig. 6i). As illustrated in Fig. 6j, GO enrichment analysis revealed the top 20 molecular function (MF) terms among the DEGs. Notably, the downregulation of G protein-coupled chemokine receptor activity exhibited the greatest statistical significance, providing compelling genetic-level evidence for the precise blockade of Cp1 against the C5a‒C5aR1 axis. A bubble map of the results of the Reactome pathway enrichment analysis in Fig. 6k also showed the most prominent alterations in the DEGs related to GPCR ligand binding, confirming the precise blockade of Cp1. The enrichment of signaling pathways in the KEGG database revealed significant overlap with key downstream signaling cascades of C5a, including the inflammatory response, apoptosis, and cell migration pathways (Fig. 6l, m). Furthermore, subcellular localization analysis highlighted the predominant presence of DEGs in the membrane-bound and extracellular compartments, suggesting that the altered gene expression may be attributed to Cp1 preincubation-mediated remodeling of extracellular receptor-associated signaling pathways (Fig. 6n). In summary, bioinformatics analysis confirmed that Cp1 treatment blocked and reshaped the C5a-induced gene expression profile in PBMCs, providing compelling transcriptome-level explanations for both the in vitro and in vivo results of this study.

### Cp1 exhibits satisfactory biosafety

The biosafety profile of the novel cyclic peptide drug Cp1 was systematically assessed through in vitro and in vivo studies. Cytotoxicity assays revealed no significant reduction in cell viability after incubation with gradient concentrations of Cp1 (Supplementary Fig. 4a). Plasma cytokine profiling (Supplementary Fig. 4b) and hematological analysis (Supplementary Fig. 4c), suggesting that no immunotoxicity or acute myelosuppression was induced by Cp1. Blood biochemical analysis (Supplementary Fig. 4d) and histopathological examination (Supplementary Fig. 4e) further verified that no hepatorenal toxicity or tissue injury was induced by Cp1. Collectively, these data demonstrated the favorable biosafety of Cp1.

## Discussion

Sepsis is a complex clinical syndrome characterized by intricate pathophysiological mechanisms and a highly dynamic clinical trajectory, which presents great challenges for its therapy.^23–25^ Excessive activation of the complement system plays crucial roles in the pathogenesis of sepsis. When selecting complement inhibitors for sepsis prophylaxis and treatment, it is essential to comprehensively consider the inhibition mechanisms, the specific pathological stage of sepsis, and existing clinical evidence. Complement inhibition has exploded during the last five years from one single C5 inhibitor to 13 FDA-approved drugs for the treatment of 8 different diseases.^26^ However, new drugs are still needed for differential treatment and to alleviate the substantial economic burden.

The C5a protein serves as an integral component of the complement system and plays multifaceted and critical roles in sepsis pathogenesis. Although C5a/C5aR signaling is essential for host defense, excessive C5a can be detrimental.^10^ Disrupting C5a‒C5aR interactions represents a promising therapeutic strategy for sepsis. Therapeutically, C5a blockade offers broader inhibition but risks immunosuppression, whereas C5aR blockade provides greater specificity yet faces limitations owing to receptor redundancy and the allosteric complexity of C5aR in drug design.^27^ From the perspective of targeting challenges, C5a neutralization requires a prolonged half-life, whereas C5aR-targeting agents present the dual challenges of tissue penetration and dynamic dose regulation.^28^ In view of this, targeted blocking of upstream C5a in the inflammatory cascade represents a strategically optimal approach to effectively block the CS with minimal drug exposure.^29,30^

A8_Δ71-73_, a C5aR1 antagonist peptide established by Magnus Otto et al., represents a milestone achievement in the field of therapeutic complement inhibition. It competitively blocks the interactions between C5a and C5aR1 by mimicking the C-terminal structure of C5a.^31^ Furthermore, crystallographic studies of A8_Δ71-73_, murine C5a, and C5a-desArg have provided a critical foundation for subsequent research on complement inhibition.^32^ Zhuang et al. further revealed that the extracellular N-terminal loop of C5aR1 is an important anchoring site for high-affinity binding to C5a.^33^ Notably, in our work, the molecular docking and homology modeling results collectively demonstrated that Cp1 triggered a dislocation of the binding anchor site between C5a and the N-terminus of C5aR1, representing a fundamentally distinct blocking mechanism of C5a‒C5aR1 interactions compared with that of A8_Δ71‒73_. The blocking specificity of Cp1 against C5a-C5aR1 was further confirmed via a competitive binding assay. The RNA-seq data of PBMCs validated the inhibitory effects of Cp1 on the C5a‒C5aR1 axis downstream signaling at the transcriptional level. However, on the basis of the present molecular docking results and competitive binding assay results, we cannot yet conclude that Cp1 does not interfere with the interaction between C5a and C5aR2. Future studies are necessary to provide more definitive evidence to fully elucidate the potential role of Cp1.

The challenges associated with developing C5a-blocking agents are due mainly to the protein’s low molecular mass, shallow surface topology and concentration-dependent multimerization at physiological concentrations.^32,34^ In our work, hemolysis assays confirmed that the binding of Cp1 to C5a does not interfere with C5 cleavage or MAC formation, whereas the weak specificity of Cp3 limits its utility. The currently approved C5-neutralizing mAb eculizumab has demonstrated clinical benefits in rare diseases.^35^ However, owing to the potential increased risk of *Neisseria meningitidis* infection associated with eculizumab, current evidence lacks large-scale randomized controlled trials (RCTs) to support its routine use in sepsis treatment.^36^

Owing to the remarkably short half-life (<5 min) of C5a, long-acting inhibitors are needed to efficiently trap and neutralize C5a.^37^ Moreover, the disordered protease milieu in septic plasma demands increased plasma stability of inhibitors to maintain therapeutic efficacy. In the present study, Cp1 exhibited superior plasma stability after cyclization, with approximately 90% of Cp1 retained in the plasma 14 days after administration. This prolonged antibody-like half-life of Cp1 ensures a sustained plasma therapeutic concentration, thereby offering significant potential for maintaining immune homeostasis in sepsis. Since the desarginated form of C5a contributes to some extent to the inflammatory response,^38^ the binding affinity data for C5a-desArg (3.58E-6) were further provided to demonstrate Cp1’s potential for effectively blocking C5a-desArg as well.

The therapeutic blockade of C5a requires disease-specific affinity optimization according to distinct pathogenic mechanisms.^30^ For autoimmune diseases, maximal binding affinity is essential. However, therapeutic agents for sepsis need to benefit from their moderate affinity for C5a, thereby sustaining microbial clearance while controlling inflammation. NOX-D20 is a PEGylated RNA/DNA hybrid aptamer inhibitor for human C5a with exceptional K_D_ performance (0.1 nM).^39^ Despite its potent pharmacological targeting of C5a, studies of NOX-D20 have not continued to be conducted in clinical trials. Prior studies reported that NOX-D20 treatment resulted in 3- to 5-fold or 2- to 3-fold reductions in the levels of inflammatory factors (IL-6 and TNF-α)/chemokines (CCL2, CXCL1 and CXCL2) in the plasma or PLF, respectively.^40^ Our findings revealed that Cp1 exhibited significantly enhanced therapeutic efficacy in CLP-induced septic mice, with 50-fold, 7.14-fold, 21.7-fold and 61.7-fold suppression of the corresponding systemic inflammatory factors/chemokines in plasma, along with 3.7-fold, 3.3-fold, 25-fold, and 4.3-fold attenuation of the corresponding local inflammatory factors/chemokines in the PLF, respectively. Encouragingly, the C5a-blocking Cp1 strain developed in this study resulted in a 300% improvement in the median survival time of septic mice compared with that of the untreated CLP group. In summary, following once administration, Cp1 establishes a robust immunomodulatory buffering system in the circulation that effectively modulates inflammatory responses without compromising host defense against secondary infections. In a subsequent assay, we further demonstrated the consistent superior response and clearance capability of Cp1 compared with those of the untreated group regardless of the duration of Cp1 exposure. The superior pathogen clearance observed in the Cp1-treated group relative to the CLP group may have resulted from the ability of Cp1 administration to effectively counteract sepsis-induced phagocytic impairment (particularly in neutrophils) mediated by excessive C5a. Given the plasma stability of Cp1 and the significantly reduced inflammatory response induced by Cp1, we speculated that Cp1 may promote bacterial clearance by alleviating immunosuppression compared with that in the untreated CLP group.

Favorable biocompatibility is essential, especially for therapeutic agents intended for sepsis prevention and therapy. PEGylation is known to induce immune responses, including anti-PEG antibody generation, complement activation and hypersensitivity reactions.^41^ The incorporation of 40kPEG in NOX-D20 may introduce immunogenicity as well as hepatorenal toxicity.^40^ However, these PEG-mediated adverse effects might be obscured by immune dysregulation and the complex pathophysiology of sepsis. In contrast, the ideal water solubility, favorable biosafety and minimal dosing frequency of Cp1 in the present study provided additional benefits to its outstanding therapeutic efficacy in sepsis.

In summary, our work developed a novel long-acting C5a-targeted blocking cyclic-peptide drug, Cp1, with a unique cyclic peptide sequence, a distinctive blocking mechanism of the C5a‒C5aR1 interaction and a cost-competitive advantage, enabling the potent suppression of hyperinflammation to maintain immune homeostasis while minimizing innate immune dysfunction and organ dysfunction. An effective therapeutic window should be further established for clinical translation. Drug combination therapy combined with supportive care may represent a promising strategy for managing sepsis with a complex etiology.

## Materials and methods

### Animals and ethical approval

All animals were purchased from GemPharmatech Co., Ltd. (Nanjing, China). All the animal studies were conducted in accordance with institutional guidelines and approved by the Ethics Committee of Changhai Hospital and Fudan University.

### Competitive binding assay by FCM

For the validation of peptide affinity for C5a. Peripheral blood neutrophils from the mice were isolated via density gradient centrifugation (Solarbio P9201, Beijing, China). A total of 10 ng of Cy5-labeled mouse C5a protein (MCE, Shanghai, China) was preincubated with linear peptides or cyclic peptides (10 or 100 ng) at room temperature for 0.5 h. Subsequently, 2×10^5^ mouse neutrophils were incubated with Cy5-labeled mouse C5a or Cy5-labeled mouse C5a/peptide complexes at 4 °C for 0.5 h in the dark, followed by centrifugation, washing with PBS and flow cytometric analysis (Sony ID7000™, Japan).

To validate the Cp1-specific blockade of C5a-C5aR1. PMNs isolated by flow cytometric sorting from Oribiotech Co., Ltd. (Shanghai, China) were preincubated with C5aR1-antibody (5 μg), C5aR2-antibody (5 μg), Cp1 (10 μg), Cp1 (10 μg)+C5aR1-antibody (5 μg) or Cp1 (10 μg)+C5aR2-antibody (5 μg) at room temperature for 0.5 h, followed by incubation with 10 ng of Cy5-labeled human C5a (MCE, Shanghai, China) at 4 °C for 0.5 h in the dark. The samples were subsequently centrifuged, washed with PBS and detected via flow cytometry. PMNs were stimulated with 10 ng/mL TNF-α for 12 hours to perform competitive binding assays under activated conditions.

### Hemolytic assay

Aliquots of mouse serum were incubated with serially diluted peptides Cp1 or Cp3 (in PBS) at 37 °C for 30 min prior to analysis. The anti-C5 antibody eculizumab (MCE, Shanghai) was selected as the positive control. Hemolysin-opsonized sheep erythrocytes (2% suspension in PBS) were added to the reaction system, followed by 0.5 h of incubation at 37 °C. Hemolytic activity was quantified by measuring the absorbance of the supernatant at 540 nm via a multimode microplate reader (BioTek Synerrgy H1, Agilent, USA).

### Chemotaxis assay

One hundred nanograms of human C5a protein (ACROBiosystems, Beijing, China) was preincubated with linear or cyclic peptides at the indicated concentrations (10 ng, 100 ng, or 1 μg) in 1640 complete culture medium for 30 min. PMNs isolated by flow cytometric sorting for chemotaxis assays were purchased from Oribiotech Co., Ltd. (Shanghai, China). A total of 1 × 10^4^ cells were added to the upper chamber of 96-well cell chemotaxis plates (Sartorius, Germany) and allowed to migrate at 37 °C for 24 h. The chemotactic response of neutrophils toward C5a with or without C5a-blocking peptides was quantified and analyzed via an IncuCyte ZOOM^®^ Live-Cell Imaging System (Sartorius, Germany). The chemotactic response of mouse neutrophils was assessed following the established protocol for PMNs.

### Capillary leakage

To evaluate microvascular leakage in the murine abdominal cavity, the mice received 100 μg of Cp1 via intravenous administration one hour after CLP surgery. Subsequently, 200 μL of 0.25% (w/v) Evans blue dye was injected intravenously for permeability quantification. Twenty-four hours later, the plasma and peritoneal lavage fluid (PLF) of the mice were collected and subjected to spectrophotometric measurement at 620 nm. To exclude hemoglobin interference, the serum absorbance values were corrected via the following formula: corrected A620 = A620 − (A405 × 0.014).^40^ Vascular permeability was quantified by the A620 ratio of PLF to serum. To assess capillary leakage in pulmonary tissues, human plasma albumin (HSA) (1 mg/75 μL) was injected intravenously after CLP surgery and Cp1 administration. Twenty-four hours later, the HSA in the bronchoalveolar lavage fluid (BALF) and plasma samples was quantified via a HAS ELISA Kit (Cusabio, Wuhan), and the lung permeability index was calculated as the ratio of the HAS in the BALF to that in the plasma.

### Plasma stability measurement and pharmacokinetic study

To evaluate the plasma stability of the peptides, 100 μg of Cp1 or K1 was incubated with 50% plasma from C57BL/6 normal mice or CLP model mice at 37 °C for 24 h.^42^ The peptides in the plasma were extracted with 15% TFA and quantified via reversed-phase high-performance liquid chromatography (HPLC) (Agilent 1260, USA). For the pharmacokinetic study, C57BL/6 mice (8–10 weeks old) were intravenously administered 100 μg of Cp1 via tail vein injection. Serial blood samples (50 μL each) were collected from the retro-orbital venous plexus at predetermined time intervals (0.083, 0.5, 1, 2, 4, 8, 11 and 24 h; 2, 3, 4, 6, 9 and 14 days post-injection) via heparinized capillaries.^43^ The plasma was immediately separated by centrifugation (3000 × *g*, 10 min, 4 °C) and stored at −80 °C until analysis.

### Inflammatory factor and chemokine determination in plasma and PLF

At 1 h post-CLP surgery, the mice received 100 μg/20 g or 300 μg/20 g Cp1 via i.v. injection. Twenty-four hours later, whole blood and PLF were collected under anesthesia. The plasma and PLF supernatants were further isolated via centrifugation (3000 × *g*, 10 min, 4 °C) and subjected to inflammatory factor and chemokine profiling via the Luminex® multiplex assay (Wayen Biotechnologies, Shanghai, China). The percent inhibition was calculated relative to that of the sham group (set as 0%).

### Determination of cfDNA and elastase in plasma

To determine the levels of cfDNA and elastase in plasma, blood samples were collected, and plasma was prepared 24 h after CLP surgery. cfDNA was extracted via the VAHTS Serum/Plasma Circulating DNA Kit (Vazyme, Nanjing) and quantified via qPCR. Elastase was quantified via an NE ELISA Kit (Cusabio, Wuhan).

### Quantification of neutrophil phagocytosis

To assess the phagocytic function of neutrophils, PMNs were stimulated with hC5a or hC5a/Cp1 complexes for 6 h, followed by incubation with 20 μL of Red *E. coli* BioParticles™ or Green *S. aureus* BioParticles™ (Invitrogen, USA) at 37 °C for 0.5 h in the dark.^44^ After being washed with precooled PBS and centrifuged (40 × *g*, 5 min), the samples were subjected to flow cytometric quantification for phagocytosis analysis.

For the live bacterial phagocytosis assay, 2 × 10^5^ PMNs were prestimulated with 100 ng of hC5a or hC5a/Cp1 complexes for 6 h. The PMNs were incubated with DMAO-labeled *E. coli* (ATCC 25922) or *S. aureus* (ATCC 29213) at an MOI (multiplicity of infection) of 10 for 0.5 h at 37 °C in the dark, followed by pretreatment with precooled gentamicin (50 μg/mL). The intracellular fluorescence intensity of PMNs was quantified via flow cytometry.

### Bacterial burden

Whole blood samples were aseptically dropped onto nutrient agar plates. Glass beads were added, and the samples were rocked horizontally to ensure the uniform distribution of blood samples. The inoculated plates were incubated at 37 °C for 24 h, after which colony-forming units (CFUs) were counted. The colonies on the culture plates were counted via ProtoCOL 3 HD (Synbiosis, UK).

### Response to secondary infection

To evaluate the impact of short-term (24 h) drug exposure on the secondary infection response. Whole blood samples were collected from each group at 24 h post-CLP surgery and stimulated with 1 μg of LPS. The IL-6 levels at 0 h, 2 h, and 4 h poststimulation were quantified via an ELISA kit.

To evaluate the impact of long-term (14 d) drug exposure on the secondary infection response. The mice were intraperitoneally injected with 3 mg/kg LPS and then given 100 μg/20 g Cp1 via the tail vein 1 h later. Fourteen days later, 1 × 10^7^ *E. coli* were intravenously injected into the mice. Blood samples were collected at 24 h post infection for bacterial load quantification. The inoculated plates were incubated at 37 °C for 24 h to count CFUs/mL in blood.

### Statistical analysis

The quantitative data are expressed as the means ± standard deviations (SDs). Two-group comparisons were performed via unpaired two-tailed Student’s *t* tests. For three or more group comparisons, one-way analysis of variance (ANOVA) followed by Tukey’s post hoc test was performed. A probability value of *p* < 0.05 was considered statistically significant for all analyses. All the statistical analyses were implemented with GraphPad Prism 10.1.2 (GraphPad Software, San Diego, CA, USA).

## Supplementary information

Supplementary Materials

## Data Availability

All data supporting the findings of this study are available in the main text and supplementary materials. The transcriptomic data of peripheral mononuclear cells have been deposited in the NCBI Sequence Read Archive (SRA) database (BioProject: PRJNA1331683).
