# Supplementary material for: A novel long-acting C5a-blocking cyclic peptide prevents sepsis-induced organ dysfunction via effective blockade of the inflammatory cascade
Source: Signal Transduct Target Ther. 2025 Nov 5;10:362. doi: 10.1038/s41392-025-02457-8 (PMC12586598; doi:10.1038/s41392-025-02457-8)
Supplement: Supplementary file 1 — Supplementary Materials [file 41392_2025_2457_MOESM1_ESM.docx]

Supplementary Materials for

A novel long-acting C5a-blocking cyclic-peptide prevents sepsis-induced organ dysfunction via effective blockade of inflammatory cascade

Zimiao Luo^1#^*, Pengfei Luo^1#^, Haoyu Gu^1^, Xiaoyan Hu^1^, Shichu Xiao^1^, Weiyue Lu^2^*, Zhaofan Xia^1^*.

* Correspondence: Zimiao Luo (luo_zimiao@163.com); Weiyue Lu (wylu@shmu.edu.cn); Zhaofan Xia (xiazhaofan_smmu@163.com).

^#^ These authors contributed equally to this work: Zimiao Luo, Pengfei Luo.

**This PDF file includes:**

Materials and Methods

Figures. S1 to S4

Materials and Methods

*In vitro selection*

Multiple distinct binding sequences were obtained based on a 12-peptide library of mouse C5a protein (1-77) (UniProt ID: P06684) using the phage display technology. Four preferred sequences K1-K4 were selected for further validation. The linear and cyclic peptides (K1, K2, K3, K4, Cp1 and Cp3) in this project were synthesized by Shanghai Science Peptide Biological Technology Co., Ltd.

Molecular docking and binding mode analysis

All molecular docking simulations were carried out using Schrödinger Maestro 12.8. The 3D structure of mouse C5a (PDB ID: 4P3A) was prepared by removing co-crystallized water molecules, adding hydrogen atoms, and optimizing hydrogen-bonding networks via the Protein Preparation Wizard. The 2D structure of Cp1 was subjected to energy minimization using the LigPrep module with the OPLS2005 force field (convergence threshold: 0.30 Å RMSD for heavy atoms) to generate optimized 3D conformations. Molecular docking was subsequently performed using the Schrödinger Maestro 12.8 in extra precision (XP) mode to predict the binding mode of Cp1 with mouse C5a. The 2D diagram was outputted using the Ligand Interaction module.

The three-dimensional structure of the mouse C5a-C5aR1 complex was predicted by homology modeling using the Swiss-Model interactive web server (https://swissmodel.expasy.org/interactive). The amino acid sequences of both mouse C5a (PDB ID: 4P3A) and C5aR1 (PDB ID: 8HQC) were submitted as input, and the "Build Model" function was executed for automated template selection and model generation. A single structural model was obtained, with PDB ID: 8HQC serving as the primary template due to its high sequence similarity and structural relevance. This predicted complex structure was subsequently used for structural alignment and further computational analyses.

Sequence alignment of mouse C5a and human C5a (UniProt ID: P01031) was performed to confirm the cross-species versatility of Cp1.

CLP-induced sepsis model

The cecal ligation and puncture (CLP) model was established in male C57BL/6 mice (9-10 weeks old). Briefly, a midline laparotomy was performed to exteriorize the cecum under general anesthesia. The cecum was ligated at the distal three-quarters using sutures. Subsequently, a single through-and-through puncture was made in the ligated segment using a 22-gaμge needle, followed by small volume of fecal content was gentle extrusion. The cecum was then repositioned within abdominal cavity, and the laparotomy incision was closed in layers. All animals were administered 1 mL of sterile saline for postoperative fluid resuscitation. Sham-operated controls underwent identical surgical procedures without cecal ligation or puncture. In this work, mice received administration via intravenous injection 1 hour after CLP surgery. The animal mortality was determined by the presence of confirmed death. The survival of each group was observed for one week and the Kaplan–Meier survival curves were drawn. Blood samples were collected 24 hours after CLP-modeling for hematological analysis (Mindray BC-60R Vet) and biochemical analysis (Mindray BS-240 Vet, China), endothelial injury analysis (Njjcbio, Nanjing) and coagulation analysis (Legene, Beijing). Subsequently, mice were sacrificed to collect the main organs for morphological and pathological analysis.

Binding affinity assay by SPR

Surface plasmon resonance (SPR) analysis was conducted using Biacore 2000 system (GE Healthcare, Germany) at 37°C in running buffer (20 mM Tris-HCl, pH 7.4, 150 mM NaCl, 5 mM KCl, 1 mM MgCl₂, 1 mM CaCl₂). The CM5 sensor chips (GE Healthcare) were activated with NHS/EDC, followed by immobilization of mouse C5a (ACROBiosystems, Beijing, China), human C5a (ACROBiosystems, Beijing, China), mouse C5 (ACROBiosystems, Beijing, China) or mouse C5a-desArg (MCE, Shanghai, China). The peptide-protein interactions were characterized using a concentration-gradient approach (low to high) with a continuous flow rate of 30 μL/min (150 s injection duration). Between analyte concentrations, the sensor surface was regenerated with 10 mM glycine-HCl (pH 2.0; 5 min treatment). Binding and dissociation constants were obtained by globally fitting the data to a 1:1 Langmuir binding model using a Biacore Insight evaluation software (Cytiva, USA).

Peptide biosafety assessment

The cytotoxic potential of cyclic-peptides was evaluated in HUVECs and RAW 264.7 cells. Cells were incubated with serially diluted Cp1 (1 pg-100 μg/mL) for 24 hours at 37°C in a 5% CO₂ humidified atmosphere, followed by cell viability assessment using CCK-8 kit (Dojindo, Japan). For systemic safety evaluation, whole blood samples were collected for hematological analysis, biochemistry analysis and inflammatory profiling analysis. Following euthanasia, major organs were harvested, fixed and processed for H&E staining.

RNA sequencing analysis

Human peripheral mononuclear cells were stimulated with 100 ng/mL of human C5a protein (MCE, China) or a complex of human C5a with Cp1 for 1 h. After RNA extraction, purification and library construction, the Next-Generation Sequencing (NGS) technology was used to perform paire-end (PE) sequencing based on Illumina sequencing platform in Bioprofile Biotechnology Co., Ltd (Shanghai, China). The expression of each gene was calculated and further subjected to differential expression analysis, enrichment analysis and cluster analysis.


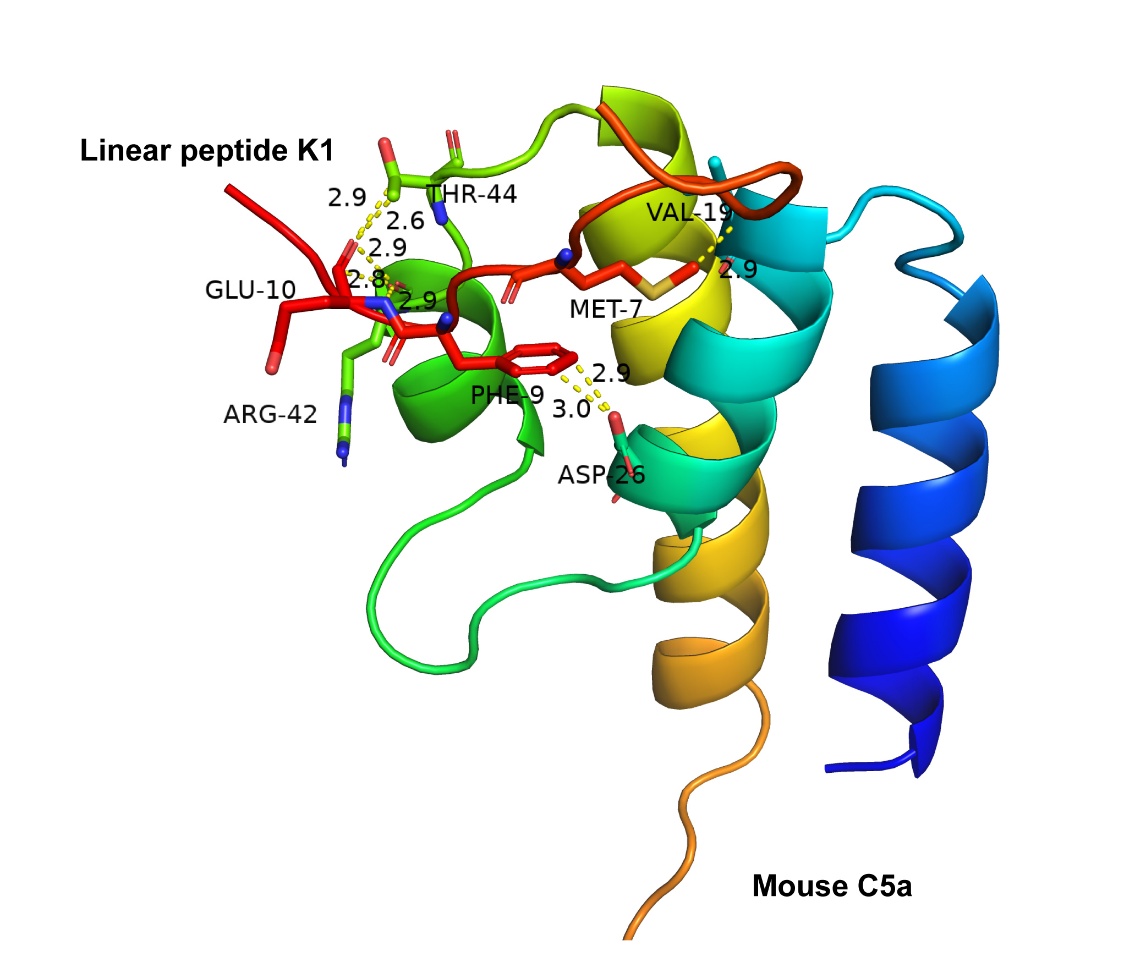


Figure. S1

**Molecular docking of linear peptide K1 with mouse C5a protein**. Cartoon diagram of the binding of linear peptide K1 to the mouse C5a protein. K1 is shown in red. Hydrogen bond interactions are shown as yellow dashed lines.


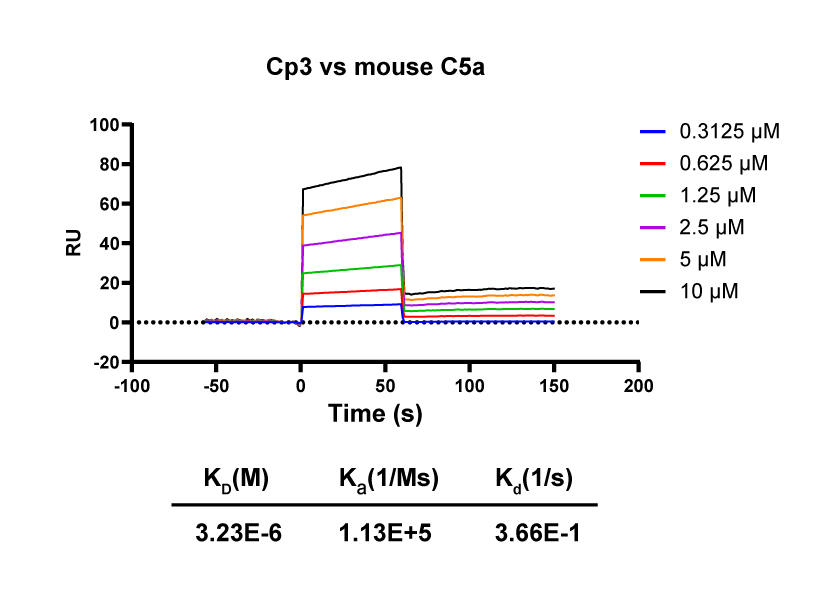


Figure. S2

**SPR analysis of cyclic peptide Cp3 with mouse C5a protein**. The binding response of immobilized mouse C5a protein to Cp3 with a Cp3 concentration gradient from 0.3125 μM to 10 μM.


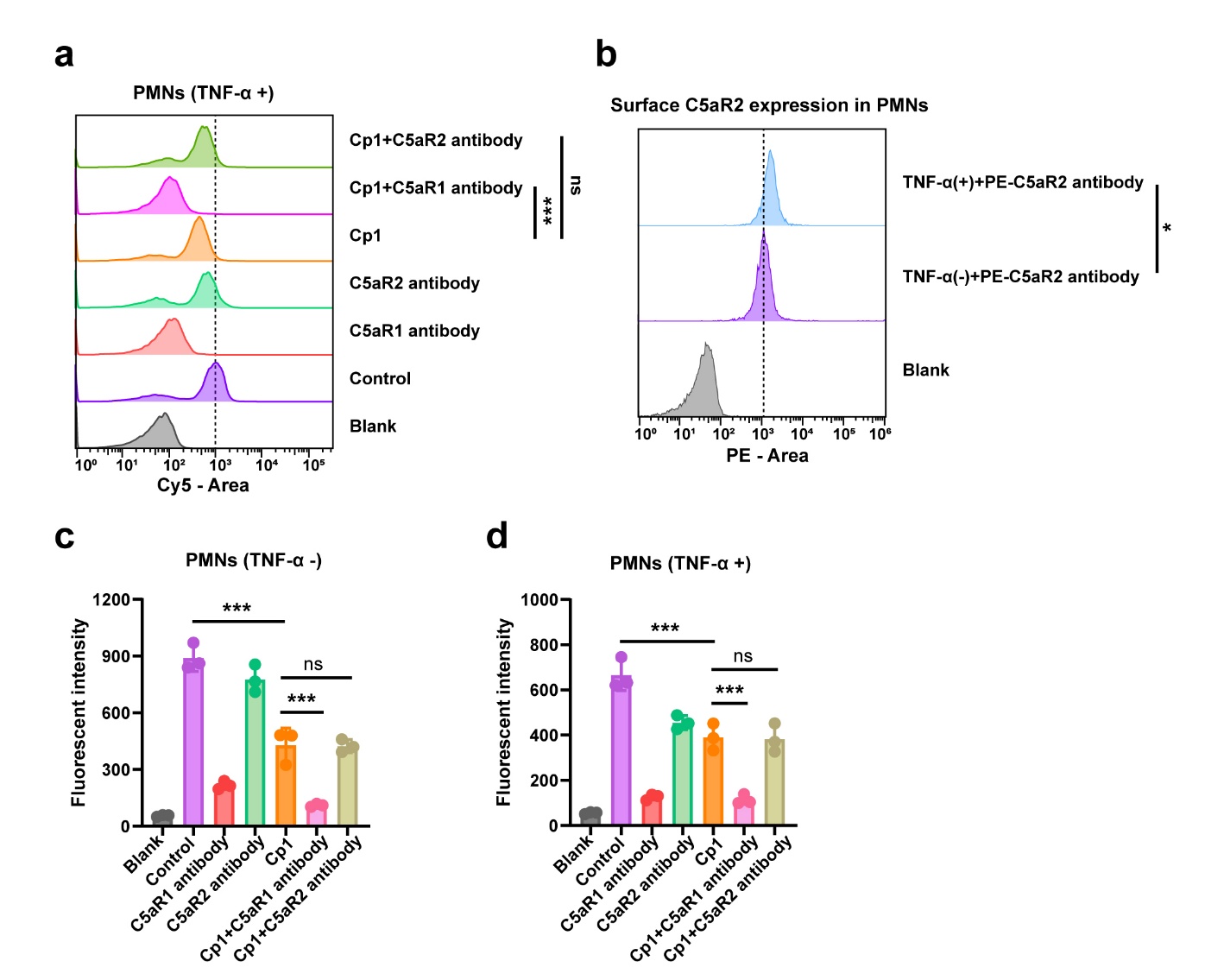


Figure. S3

**Validation of Cp1-specific blockade of C5a-C5aR1**. **a** PMNs (with TNF-α stimulation for 12 h) were preincubated with C5aR1-antibody, C5aR2-antibody, Cp1, Cp1+C5aR1-antibody or Cp1+C5aR2-antibody at room temperature for 0.5 h, followed by incubation with Cy5-C5a at 4°C for 0.5 h in the dark. The Cy5-C5a signals were detected by flow cytometry. *n* = 3. **b** The surface C5aR2 expression levels in PMNs with or without TNF-α stimulation (12 h) were detected by flow cytometry. *n* = 3. **c** The Cy5 semi-quantitative results of PMNs (without TNF-α stimulation) after the treatment in Fig. 2h, *n* = 3. **d** The Cy5 semi-quantitative results of PMNs (with TNF-α stimulation for 12 h) after the treatment in Fig. S3, *n* = 3. The data are presented as the means ± SDs. **P* < 0.05, ***P* < 0.01, ****P* < 0.001.


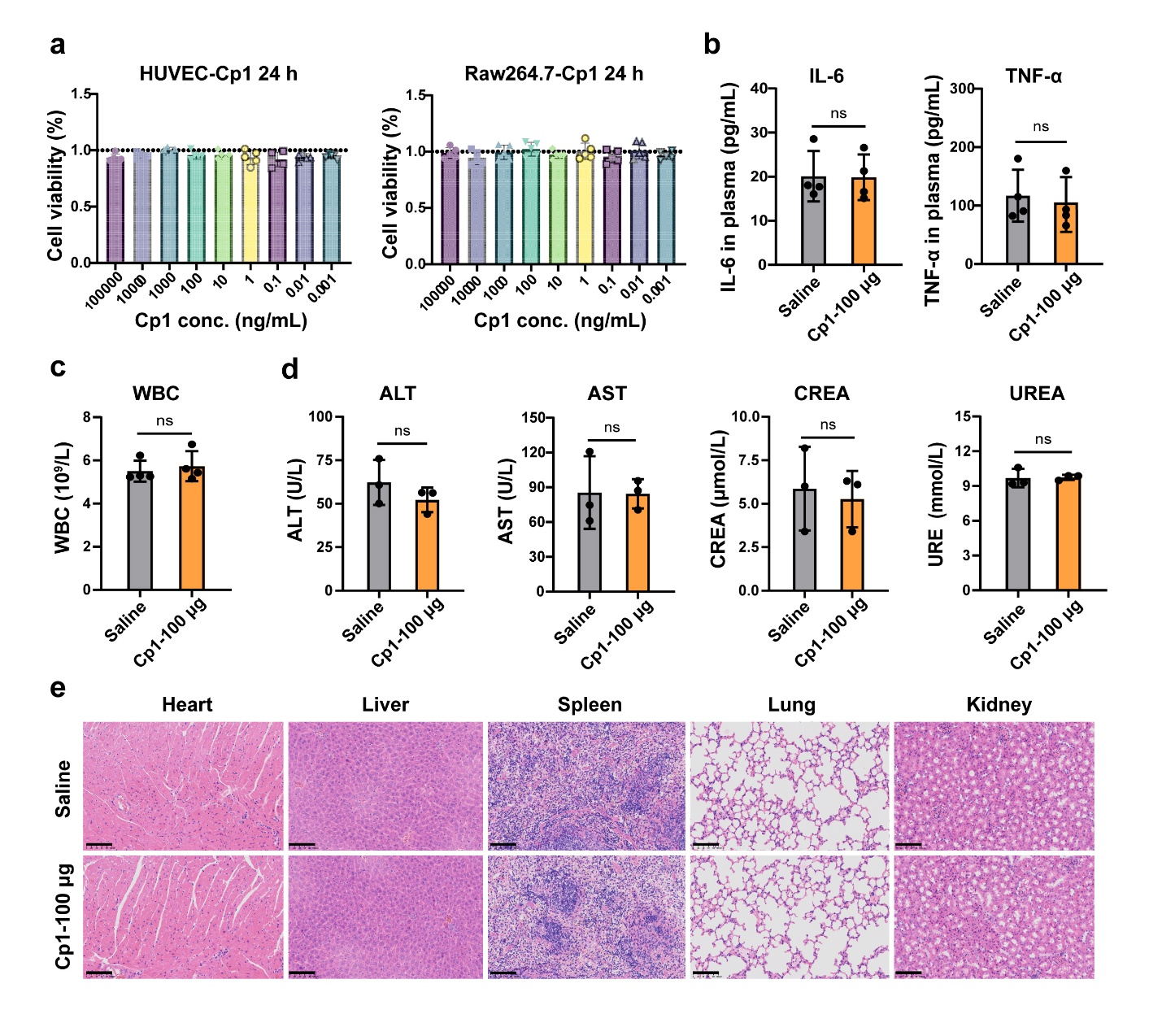


Figure. S4

**Biosafety evaluation of Cp1. a** The cell activity of HUVEC and Raw264.7 cells incubated with different concentrations of Cp1, *n* = 6. **b** The immunotoxicity analysis of Cp1 in normal mice 24 h after intravenous administration of Cp1 (100 μg/20 g), *n* = 4. **c** The liver or renal function indexes in normal mice 24 h after intravenous administration of Cp1 (100 μg/20 g), *n* = 4. **d** The hematological analysis of normal mice 24 h after intravenous administration of Cp1 (100 μg/20 g), *n* = 4. **e** The H&E staining results of pathological sections from major organs collected 24 h after intravenous administration of Cp1 (100 μg/20 g), *n* = 3. The scale bar represents 100 μm. The data are presented as the means ± SDs. **P* < 0.05, ***P* < 0.01, ****P* < 0.001.
